# Sexual Dysfunction in Women with Cancer: A Systematic Review of Longitudinal Studies

**DOI:** 10.3390/ijerph191911921

**Published:** 2022-09-21

**Authors:** Thais Sousa Rodrigues Guedes, Marcello Barbosa Otoni Gonçalves Guedes, Rebeca de Castro Santana, José Felipe Costa da Silva, Amanda Almeida Gomes Dantas, Mirari Ochandorena-Acha, Marc Terradas-Monllor, Javier Jerez-Roig, Dyego Leandro Bezerra de Souza

**Affiliations:** 1 Graduate Program in Health Science, Center of Health Science, Federal University of Rio Grande do Norte (UFRN), Campus Universitário Lagoa Nova, Natal 1524, Brazil; 2 Department of Physiotherapy, Federal University of Rio Grande do Norte (UFRN), Campus Universitário Lagoa Nova, Natal 1524, Brazil; 3 Department of Public Health, Graduate Program in Health Science, Federal University of Rio Grande do Norte (UFRN), Campus Universitário Lagoa Nova, Natal 1524, Brazil; 4 Research Group on Methodology, Methods, Models and Outcomes of Health and Social Sciences (M_3_O), Faculty of Health Sciences and Welfare, Centre for Health and Social Care Research (CESS), University of Vic-Central University of Catalonia (UVic-UCC), C. Sagrada Família, 7, 08500 Vic, Spain

**Keywords:** neoplasms, sexual dysfunction, women, systematic review, longitudinal studies

## Abstract

Background: Several factors affect sexual function, including cancer development and treatment. This study summarized the risk of women with cancer of developing sexual dysfunctions. Methods: This systematic review was conducted according to the Preferred Reporting Items for Systematic Reviews and Meta-Analyses (PRISMA). We searched the EMBASE, PubMed, LILACS, SciELO, CINAHL, Scopus, and Web of Science databases using the descriptors cancer, neoplasms, sexual dysfunction, sexual function, and women. The Quality Assessment Tool for Observational Cohort and Cross-Sectional Studies assessed the quality of studies. Results: Sixteen studies were included in this review. Women with cancer presented sexual dysfunctions in 14 out of 16 included studies. The incidence of sexual dysfunctions ranged from 30% to 80%, while the risk of developing sexual dysfunction increased 2.7- and 3.5-fold in women with cervical and breast cancer, respectively. Conclusion: Different cancer treatments increase the risk of developing sexual dysfunction in women, especially desire, arousal, and orgasm, leading to biopsychosocial changes in the health of this population.

## 1. Introduction

The increase of survivors of cancer in recent decades has drawn attention to factors previously neglected in the health of these individuals, such as sexual function. Specifically, the female sexual function is highly impacted by health-related events, such as cancer, triggering sexual dysfunctions and reducing quality of life [1,2].

Sexual dysfunctions are characterized by persistent and recurrent difficulties in accessing and completing one or more phases of the physical sexual response (i.e., desire, arousal, orgasm, and resolution) [3,4]. These dysfunctions are also affected by biological, psychological, behavioral, and sociocultural factors and can be classified as hypoactive sexual desire, dyspareunia, and arousal orgasmic disorder [5,6]. In this sense, sexual function can be directly or indirectly impacted by pregnancy, alcohol, or nicotine consumption, pelvic organ prolapse, urinary incontinence, post-menopause, stress, mood disorders, body image problems, low self-esteem, feelings of inadequacy, age, level of education, quality of long-term relationship with the partner, chronic or neurological diseases, and side effects of cancer treatment (adjuvant or neoadjuvant) [3,6,7].

Cancer diagnosis and treatment may also cause suffering in women, who are usually vulnerable [8]. Most women with breast cancer have negative feelings and psychopathological, biological, and social symptoms, such as denial, anger, fear, fatigue, and mood, sleep, and sexual disorders [2]. Moreover, cancer treatment involves surgical interventions, neoadjuvant chemotherapy, anti-hormonal therapy, radiation, or targeted therapies, which may result in psychosocial effects and sexual dysfunctions [9].

Although associations between different cancers and sexual dysfunctions in women have been studied worldwide [10,11,12], controversial findings and the heterogeneity of methods and populations highlight the need to systematize data. Furthermore, understanding the impacts of cancer on sexual function may help governments to develop public health policies and instruct healthcare professionals to provide better clinical decisions and accurate assessments and interventions for this population.

To our knowledge, systematic reviews about sexual dysfunction in women with cancer focused mainly on the prevalence and included cross-sectional or mixed studies [13,14]. Additionally, the literature lacks systematic reviews considering only longitudinal studies estimating the risk of developing sexual dysfunctions. Therefore, this systematic review aimed to answer the following questions: are adult women who experienced cancer treatment at greater risk for sexual dysfunction? What is the incidence of sexual dysfunctions in this population? What are the main risk factors for sexual dysfunctions associated with cancer in women?

## 2. Materials and Methods

### 2.1. Study Design and Protocol Registration

We conducted a systematic review according to the Preferred Reporting Items for Systematic Reviews and Meta-Analyses (PRISMA, 2020). The study was registered in the International Prospective Register of Systematic Reviews (PROSPERO, no. CRD42021115580).

### 2.2. Search Strategy

An extensive search was performed without a publication date restriction in the following databases: PubMed, LILACS, SciELO, CINAHL, Scopus, Web of Science, and EMBASE. The same researchers conducted the search strategy in January 2019 and updated in September 2021 by grouping terms in English, Portuguese, and Spanish to optimize sensitivity and precision (Table 1).

### 2.3. Inclusion and Exclusion Criteria

Inclusion criteria were longitudinal prospective or retrospective studies conducted with women aged 18 years diagnosed with (treated or untreated) cancer (any anatomical location), and that assessed sexual dysfunction as a primary or secondary outcome. We excluded studies published as full reports, abstracts, letters to the editors, comments, reviews, studies with women who had sexual dysfunction before cancer diagnosis, studies without a control group, those with a sample not representative of the population (i.e., without different ethnicities and sexual minorities), and those that did not use specific instruments to assess sexual dysfunction.

Two blinded researchers (RCS and JFCS) independently reviewed the titles and abstracts of studies according to inclusion and exclusion criteria. The remaining studies were read in full to verify the eligibility. In case of disagreement, a third reviewer (TSRG) assessed the eligibility of the study.

### 2.4. Data Extraction

Two pairs of blinded researchers (RCS and JFCS; TSRG and AAGD) independently extracted and recorded data from studies using a standardized data extraction form based on inclusion criteria. Data extracted were author, year of publication, country, study design, number of participants, age, marital status, level of education, cancer treatment, follow-up, instruments to assess sexual dysfunctions, incidence, cut-off points for classifying sexual dysfunctions, domains of sexual function affected, other symptoms, outcomes, percentage of sexually active women, reasons for sexual inactivity, and impacts of treatment on sexual function.

### 2.5. Quality Assessement

The quality of studies was assessed using the Quality Assessment Tool for Observational Cohort and Cross-Sectional Studies, developed in 2013 to help researchers focus on essential concepts for the internal validity of a study (NHLBI, 2021). This assessment was performed by two independent researchers (MTM and MOA), and disagreements were evaluated by a third researcher (TSRG). The authors of the included studies were consulted in case of insufficient data.

## 3. Results

**Figure 1:**
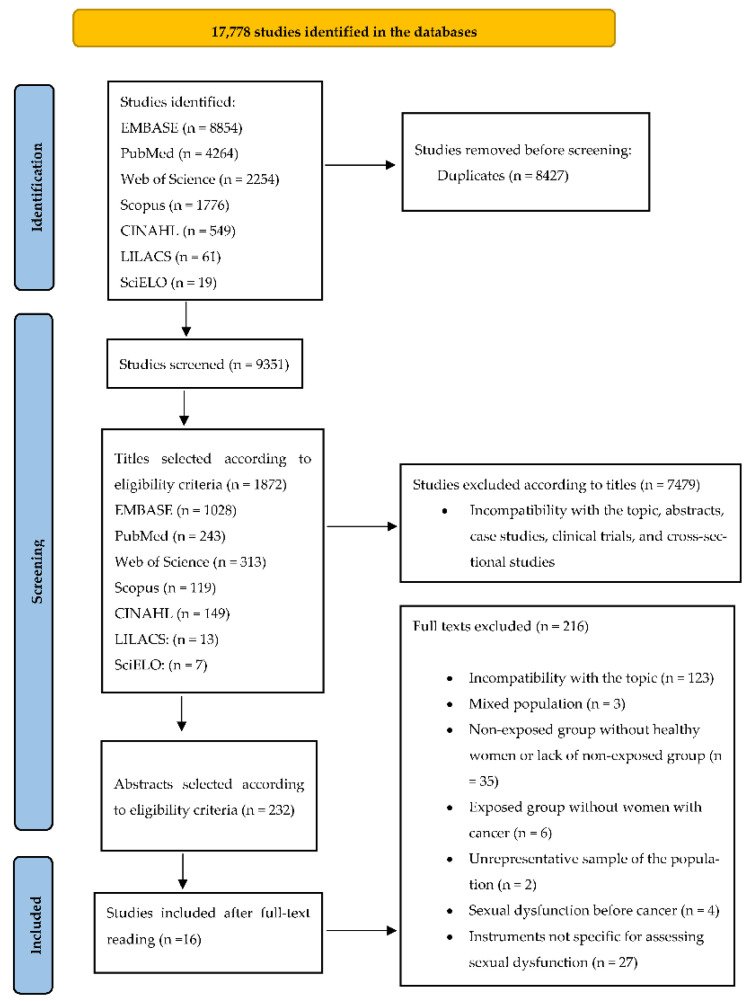
PRISMA 2020 flow diagram.

We found 17,778 studies (EMBASE: 8854; PubMed: 4264; Web of Science: 2254; Scopus: 1776; CINAHL: 549; LILACS: 61; and SciELO: 19); however, 8427 were duplicated. We also excluded 9109 studies according to eligibility criteria for titles and abstracts. Of the remaining 232 studies, 216 were excluded after full-text reading (Figure 1). Therefore, 16 studies were included in this systematic review.

Table 2 presents the descriptive characteristics of the included studies. The age of women ranged from 25 to 69 years [1,2,9,15,16,17,18,19,20,21,22,23,24,25,26,27]; most were married [1,2,9,18,20,21,24,25,27] and lived in the United States, Germany, or Belgium [9,15,16,17,18,20,21,23,25]. The types of cancers analyzed in women with sexual dysfunctions were breast, cervical, endometrial, ovarian, vulvar, and gynecological [1,2,9,15,16,17,18,19,20,21,22,23,24,25,26,27]. Additionally, women with cancer were treated with surgery, chemotherapy, radiotherapy, or hormonal therapy [1,2,9,15,16,17,18,19,20,21,22,23,24,25,26,27]. The follow-up time ranged from six months to five years [16,17,18,19,20,21,22,23,25].

Women with cancer presented sexual dysfunctions in almost all included studies, except for two [1,16]; the incidence ranged from 30% to 80% [15,19,23,26]. On the other hand, the risk of developing this condition was described in two studies and ranged from 2.7- to 3.5-fold compared with women without cancer [17,21]. Other outcomes assessed were quality of life [9,17,19,20,25], anxiety [2,19,21,27], depression [2,18,20,21,25,27], body image [21,25], urinary and bowel function [15,26], prolapse [15], sleep [2], and menopausal symptoms [1]. Most women were sexually active [9,15,16,19,21,22,25,26,27], whereas sexual inactivity was mainly due to lack of partner or sexual interest [9,15,26]. The most frequent sexual dysfunctions were reduced desire, arousal, and orgasm [2,17,19,20,23,24,25,26,27]. Moreover, most symptoms reported were dyspareunia [15,17,18,23,25], vaginal stenosis [22,26,27], fecal incontinence [16,26], abdominal pain [18,20,25], vaginal aspects [18,25], urinary incontinence [16], bleeding, hematuria, diarrhea [26], pelvic symptoms [1], menopausal symptoms [21], sexual preoccupation [19], and depression [16]. Of the 16 included studies, nine reported that cancer treatment affected sexual function [9,16,18,19,20,21,22,24,25,27]. The instruments for assessing sexual dysfunctions varied between studies, but the most used was the Female Sexual Function Index [16,17,18,26] (Table 3).

Most studies presented moderate methodological quality: eleven scored between 8 and 10 [1,9,15,16,17,18,19,20,21,22,23], and five scored ≤ 7 [2,24,25,26,27] (Table 4).

## 4. Discussion

According to the included studies, women with cancer were 2.7- to 3.5-fold more likely to develop any sexual dysfunction. Cervical, breast, and endometrial cancers were the most associated with sexual dysfunctions. The most recurrent sexual dysfunctions were reduced desire, arousal, and orgasm, while the most common psychological aspects were depression, anxiety, and body image problems. Moreover, the main social and behavioral aspects associated with sexual dysfunctions in women with cancer were age > 50 years, high levels of education (high school and higher education), and marital status. Most women were married and reported low-quality relationships with partners. Additionally, most studies presented moderate methodological quality.

### 4.1. Instruments Used to Assess Sexual Dysfunctions

Several instruments are used to assess sexual dysfunctions in women, justifying the high heterogeneity observed in our study. The most used instruments were the Female Sexual Function Index [9,19,26], Derogatis Sexual Functioning Inventory [9,23,27], Short Sexual Functioning Scale [18,19,20], and Sexual Function-Vaginal Changes Questionnaire [17,22].

A review conducted with survivors of breast cancer demonstrated that several instruments could assess sexual function at baseline and after treatment, detecting the main early symptoms of sexual dysfunctions and improving the quality of life of women and partners [28].

### 4.2. Incidence of Sexual Dysfunctions in Women with Cancer

Sexual dysfunctions are associated with several cancers, as demonstrated in most studies included in this review [2,16,18,19,21,22,24,25,27]. Additionally, the incidence ranged between 30% and 80% [17,19,23,26] and varied according to different regions. Jing et al. (2019) [29] observed an incidence of sexual dysfunctions of 82.8% in a study with 2684 survivors of breast cancer in Mainland China; values were slightly higher than in other countries (incidence of 72.1%).

### 4.3. Sexual Dysfunctions and Cancer

Surgery, chemotherapy, radiotherapy, and hormonal therapy are indicated to treat cancers; however, they may cause nausea, vomiting, fatigue, alopecia, weight gain, pallor, induced menopause, gynecological complaints, and sexual dysfunctions [30,31]. Physiological changes resulting mainly from systemic treatments may lead to reduced lubrication and sexual desire [21,24]; thus, the anatomical location of the tumor would not be the only cause of sexual dysfunction in this population. The subtypes of sexual dysfunction, such as reduced sexual desire [2,17,19,20,21,23,24,25,26,27], low arousal [2,17,20,25,26], and difficulty in orgasm [2,20,25,26], also interfere with the quality of the sexual activity. Although these dysfunctions were prevalent in the included studies, other dysfunctions (e.g., dyspareunia, low lubrication, and sexual satisfaction) should also be considered in clinical evaluations.

The type of cancer may influence symptoms of sexual dysfunctions in women during or after treatment [32]. Women with cervical cancer presented a 2.7-fold higher risk of developing sexual dysfunctions than women without cancer [17], mainly reduced lubrication (7.6-fold; 95%CI: 3.2–18.1) [17,22,26], dyspareunia (4.8-fold; 95%CI: 1.4–16.6) [17], satisfaction (2.1-fold; 95%CI: 1.3–3.5) [17], orgasm (1.5-fold; 95%CI: 1.1–2.2) [17], and reduced arousal and desire [17,19,26,27].

Women with breast cancer had a 3.5-fold higher risk of developing altered sexual function, mainly body image problems, than women without cancer [21,25]. The self-perception of women may also change during cancer treatment due to procedures that change body shapes (e.g., surgery) [33].

Endometrial cancer causes morphofunctional changes in sexual function, such as urinary and fecal incontinence [16], dyspareunia, and changes in vaginal aspects [18]. Cianci et al. (2020) [34] observed similar results in a study performed with 118 patients of endometrial cancer: 55.9% reported not having sexual relations with partners after surgery due to perceived changes in their bodies.

In this context, healthcare professionals are essential for mitigating the diverse symptoms caused by cancer and providing educational and multidisciplinary approaches focused on assessing and treating the sexual function of patients and partners [35].

### 4.4. Other Risk Factors for Sexual Dysfunctions

Changes in mental health (e.g., depression, anxiety, suicide, neurocognitive, and sexual dysfunctions) are common in patients with cancer [36]. However, the healthcare team routinely neglects interventions targeting mental health during and after cancer treatment in women [37]. In this review, depression [2,18,20,21,25,27], followed by anxiety [19,21,27] and impaired body image [21,25], were the most observed psychosexual aspects in women with cancer.

Mental health disorders may also interfere with sexual function and impair the lives of women under cancer treatment [38]. After treatment, women reported doubts about the disease, fear of recurrence, lack of information about treatments and follow-up, changes in lifestyle, recurrence of symptoms, and concerns about disease prevention in first-degree relatives; these doubts therefore generated psychological distress [39].

Depression is one of the major mental health disorders associated with breast cancer in women. A review found that approximately 32.2% of women evaluated had some depressive symptom associated with cancer [40]; anxiety was also recurrent. Another systematic review with 16,298 women with breast cancer reported that 41.9% of women had anxiety, considered an important risk factor for increasing the illness and suffering of women, partners, and family members [41]. Thus, according to the studies assessed, depression and anxiety increased the risk of sexual dysfunctions.

Body image problems are also common in women with cancer since treatments may change their appearance (e.g., breast asymmetry and changes in skin texture and sensitivity) [42]. Self-perceived body image is based on socially acceptable ideals of beauty and concerns regarding the reaction of society towards appearance and may lead to self-image disorders [42]. Campos et al. (2022) identified that problems with self-perceived body image were inversely associated with satisfaction with sexual life and quality of life [43]. A cross-sectional, comparative, and controlled study with 90 women with breast cancer, aged 18 to 65 years, and who underwent mastectomy and breast reconstruction reported better sexual function and body image and fewer depressive symptoms than women with isolated mastectomy [44].

Social determinants of health, such as age, education, and marital status, were also considered risk factors for sexual dysfunctions in women with cancer. We observed a wide age range (25 to 69 years) in women in the included studies, probably because of different types of cancer, regional location, and socioeconomic status. Cervical cancer is often diagnosed in women aged approximately 53 years; however, a global study observed that this cancer affects women under 45 years in 146 (79%) of 185 countries [45].

The age at which cancer is diagnosed and treated interferes with sexual and mental health of women of reproductive age [46]; therefore, understanding how age correlates with other risk factors is essential for effective interventions. Cancer diagnosis and treatment may cause different sexual dysfunctions in women at different ages, depending on lifestyle, functional capacity, and social and professional engagement [38]. Younger women with some types of cancer may also present impaired self-perceived body image and a high prevalence of sexual dysfunctions, possibly due to psychological consequences [47].

We observed that most women were married [1,9,17,18,19,20,21,22,24,25,26]. This aspect can be beneficial, considering that marriage may influence the likelihood of receiving definitive therapy and that women are less likely to die as a result of their cancer [48]. Moreover, an active marriage is associated with fewer sexual dysfunctions, early diagnosis, and effective treatments in women with cancer. On the other hand, results suggested that widowed women had an increased risk of mortality [44,49].

Low levels of education are also associated with sexual dysfunctions in women with cancer, which may delay diagnosis, increase the risk of aggressive cancer, and reduce survival [50]. In this review, most women diagnosed with cancer had high school or higher education, which may improve socioeconomic aspects and quality of life. Low levels of education, lack of knowledge regarding body structures, and health conditions were associated with increased sexual dysfunctions in women with cervical cancer [51]. Thus, a high level of education may be associated with early cancer diagnosis and a better understanding of the disease, treatment, and post-treatment [50].

The absence of a partner was the main cause of sexual inactivity in women with cancer [9,15,26]. Previous studies [52,53] showed that support, empathy, and relationship quality were important predictors of good sexual function in women after a mastectomy. Similarly, Aertes et al. (2015) [18] showed that a good quality of partner relationship decreased the chances of reporting problems with sexual arousal and orgasm in women with endometrial cancer.

The quality of relationship also affected the sexual function of women with vulvar cancer [20] and was negatively correlated with impaired arousal and orgasm, profound dyspareunia, and abdominal pain during intercourse. Additionally, a study showed that the duration of marriage impacted sexual function; the more recent the marriage, the better the sexual function of women with breast cancer [24].

Thus, we suggest the risk factors described in this review should be included as confounding or control variables in future studies assessing cancer as a risk for sexual dysfunctions.

### 4.5. Critical Analysis of the Quality of Studies

Of the 14 items used to assess the quality of studies, the justification for the sample size was the most absent; only one study provided justification for the sample size [15], hindering data extrapolation to the general population with cancer. Moreover, six studies [2,17,24,25,26,27] received “no” in the item regarding sufficient a timeframe to see an effect, which assesses whether the effect was a result of the exposure. A further six studies did not report losses during follow-up [16,17,18,19,20,22], an important factor in cohort studies since it is part of the analysis of the exposure effect. Additionally, the control of confounding variables, which reduces the influence of these variables in the analysis of outcomes, was not performed by six studies [2,15,20,22,24,25]. Although these factors were scored negatively, most items received a positive score, highlighting the quality of studies.

### 4.6. Limitations of the Study

The studies included in this review were predominantly conducted in developed countries; thus, researchers from countries with different socio-cultural characteristics should use these findings cautiously. Additionally, the studies assessed sexual dysfunctions using different instruments, criteria, and methodologies, which hindered a meta-analysis. Furthermore, older studies may not have considered the evolution of cancer treatments, which would have impacted the outcome assessed in this review. Although studies of moderate quality were retrieved from different databases, some relevant studies may not have been included.

## 5. Conclusions

The incidence of sexual dysfunctions ranged between 30% and 80% in women with cancer. Women with cancer had a 2.7- and 3.5-fold higher risk of developing sexual dysfunctions, mainly alterations in desire, arousal, and orgasm, than women without cancer. Moreover, treatments for different cancers may have led to biological, psychological, and social consequences on the health of women. In addition, depression, anxiety, and body image problems were prevalent in women with cancer and sexual dysfunctions, while the social determinants of health impacted the risk of developing sexual dysfunctions in this population.

Although cancer directly impacts several domains of the lives of women, some aspects may be neglected during or after treatment. Sexual dysfunctions, for example, should not be overlooked since they may lead to suffering and vulnerability in this population. Monitoring these women by focusing only on biological aspects of the disease without considering psychosexual issues and social determinants may result in insufficient interventions. In this context, multidisciplinary actions, health education, and social support network may be a proactive strategy for these women during and after cancer treatment. Therefore, our results may guide future public health policies related to early diagnosis, effective treatments, and cancer prevention in women. We suggest future studies assessing the evolution of cancer treatments over the years and their impact on female sexual function.

## Figures and Tables

**Table 1:** Search strategy used in the systematic review. Source: authors.

Databases	Language	Descriptors	Strategy
PubMed	English	Cancer, neoplasmas, “Sexual Dysfunction”, “sexual function” and women	(Cancer OR Neoplasms) AND (Sexual Dysfunction OR Sexual Function) AND (Women)
LILACS	English, Portuguese, and Spanish	Cancer, neoplasmas, “Sexual Dysfunction”, “sexual function” and women Câncer, neoplasia, Dysfunção sexual, Função Sexual e mulheres Cáncer, neoplasias, disfunciones sexuales, función sexual, Mujeres	(Cancer OR Neoplasms) AND (Sexual Dysfunction OR Sexual Function) AND (Women) (Neoplasia OR Câncer) AND (Disfunção Sexual OR Função Sexual) AND (Mulheres) (Neoplasias OR Cáncer) AND (Disfunciones Sexuales OR Función Sexual) AND (Mujeres)
SciELO	English, Portuguese, and Spanish	Cancer, neoplasmas, “Sexual Dysfunction”, “sexual function” and women Câncer, neoplasia, Dysfunção sexual, Função Sexual e mulheres Cáncer, neoplasias, disfunciones sexuales, función sexual, Mujeres	(Cancer OR Neoplasms) AND (Sexual Dysfunction OR Sexual Function) AND (Women) (Neoplasia OR Câncer) AND (Disfunção Sexual OR Função Sexual) AND (Mulheres) (Neoplasias OR Cáncer) AND (Disfunciones Sexuales OR Función Sexual) AND (Mujeres)
CINAHL	English	Cancer, neoplasmas, “Sexual Dysfunction”, “sexual function” and women	(Cancer OR Neoplasms) AND (Sexual Dysfunction OR Sexual Function) AND (Women)
Scopus	English	Cancer, neoplasmas, “Sexual Dysfunction”, “sexual function” and women	(Cancer OR Neoplasms) AND (Sexual Dysfunction OR Sexual Function) AND (Women)
Web of Science	English	Cancer, neoplasmas, “Sexual Dysfunction”, “sexual function” and women	(Cancer OR Neoplasms) AND (Sexual Dysfunction OR Sexual Function) AND (Women)
EMBASE	English	Cancer, neoplasmas, “Sexual Dysfunction”, “sexual function” and women	(Cancer OR Neoplasms) AND (“Sexual Dysfunction” OR “Sexual Function”) AND (Women)

**Table 2:** Descriptive characteristics of included studies.

Study	Country	Study Design	Cancer Type	Number of Participants	Age (Mean)	Marital Status	Level of Education	Cancer Treatment	Follow-Up Period
Baessler, K.; 2021 [15]	Germany	Retrospective cohort	CC	221	LARVHG: 43 VALRRG: 45 TMMRG: 51 CG: 46 UGG: 51	NR	NR	Surgery or radiotherapy	NP
İzci, F.; 2020 [2]	Turkey	Prospective cohort	BC	108	CaG: 53 CG: 52.5	43% married	40% middle or high school	Surgery, radiotherapy, chemotherapy, or hormonal therapy	NR
Mayer, S.; 2019 [9]	Germany	Retrospective cohort	BC and OC	305	BC: 56 OC: 53 CG: 46	BC: 68.9% OC: 62.0% CG: 65% Married	NR	Surgery, chemotherapy, or hormonal therapy	NP
Buckingham, L.; 2019 [16]	United States	Prospective longitudinal	EC	425	CaG: 63 CG: 57	NR	NR	Surgery, radiotherapy, or chemotherapy	Five years
Heinzler, J.; 2018 [17]	Germany	Case-control	CC	166	CaG I: 35.9 CaG II: 34.1 CG: 30.9	CaG I: 77% CaG II: 73% CG: 58% In a relationship	CaG I: 72% CaG II: 70% CG: 69% Completed high school	Surgery or chemotherapy	Six months
Soldera, S.V.; 2018 [1]	Canada	Prospective cohort	BC	407	CaG: 62 CG: 69	CaG: 64% CG: 69% Married	NR	Surgery, chemotherapy, or hormonal therapy	NP
Corrêa, C.S.L.; 2016 [26]	Brazil	Case-control	CC	74	CaG: 51.2 CG: 50.5	CaG: 51.4% CG: 73% Had a partner	CaG: 54.1% CG: 62.2% Low schooling level	Surgery, radiotherapy, or chemotherapy	NP
Aerts, L.; 2015 [18]	Belgium	Prospective longitudinal	EC	252	CaG: 62.9 BeG: 53.3 CG: 59.9	CaG: 71% BeG: 86% CG: 69% Cohabitation or married	Gca: 40% BeG: 43% CG: 77% ≥Bachelor	Surgery	Two years
Aerts, L.; 2014 [20]	Belgium	Prospective longitudinal	BC	230	GTC: 57.2 GM: 54.5 CG: 56.1	GTC: 80% GM: 83% CG: 79% Cohabitation or married	GTC: 46% GM: 43% CG: 27% ≥ Bachelor	Surgery, radiotherapy, chemotherapy, or hormonal therapy	One year
Froeding, L.P.; 2014 [19]	Denmark	Prospective longitudinal	CC	80	RVTG: 29 RAHG: 42 CG: 28.5	RVTG: 72% RAHG: 88% CG: 80% Had a partner	RVTG: 72.2% RAHG: 81.2% CG: 50% Higher education	Surgery	One year
Aerts, L.; 2014 [20]	Belgium	Prospective longitudinal	VC	58	VC: 57.38 CG: 55.28	VC: 65% CG: 73% Cohabitation or married	VC: 31% CG: 55% ≥ Bachelor	Surgery	One year
Juraskova, I.; 2013 [27]	Australia	Prospective longitudinal	CC and EC	165	CaG: 50.9 BeG: 46.9 PIG: 28.1	CaG: 72% BeG: 63% Married PIG: 71% other	CaG: 57% ≤ High school BeG: 38% PIG: 42% Higher education	Surgery, radiotherapy, chemotherapy, or brachytherapy	Six months
Pérez, M.; 2010 [21]	United States	Prospective longitudinal	BC	1033	DCIS: 57.0 Stage I: 59.1 Stage IIA: 54.5 CG: 56.5	DCIS: 63.1% Stage I: 62.3% Stage IIA: 57.5% CG: 65.6% Married	DCIS: 71% Stage I: 65% Stage IIA: 76.2% CG: 74.9 > High school	Surgery, radiotherapy, chemotherapy, or hormonal therapy	Two years
Abasher, S.M.; 2009 [24]	Sudan	Prospective cohort	BC	200	CaG: 45% aged 25 to 39 years CG: 44% aged 40 to 49 years	CaG and CG: 100% married	CaG: 32% Elementary school CG: 30% High school	Surgery, radiotherapy, chemotherapy, or hormonal therapy	NP
Jensen, P.T.; 2003 [22]	Denmark	Prospective longitudinal	CC	354	CaG: 55 CG: 55	CaG: 64% CG: 75% Had a partner	NR	Surgery, radiotherapy, or chemotherapy	Two years
Andersen, B.L.; 1989 [23]	United States	Prospective longitudinal	GyC	122	CaG: 42 CG: 39 BeG: 42	NR	NR	Surgery, radiotherapy, or chemotherapy	One year

CaG: cancer group; CG: control group; BC: breast cancer; OC: ovary cancer; CC: cervical cancer; EC: endometrial cancer; GyC: gynecological cancer; VC: vulvar cancer; NR: not reported; NP: not performed; BeG: benign group; PIG: pre-invasive group; LARVHG: laparoscopically-assisted radical vaginal hysterectomy group; VALRRG: vaginally-assisted laparoscopic or robotic radical hysterectomy group; TMMRG: laparoscopic total mesometrial resection group; UGG: urogynecological group; RVTG: radical vaginal trachelectomy group; RAHG: radical abdominal hysterectomy group; DCIS: ductal carcinoma in situ.

**Table 3:** Analysis of sexual dysfunction and repercussions.

Study	Assessment Instrument	Sexual Dysfunctions Assessment	Sexual Dysfunctions	Incidence of Sexual Dysfunctions	Risk of Sexual Dysfunctions (R2)	Domains of Sexual Function Affected	Sexually Active Women	Reason for Sexual Inactivity
Baessler, K.; 2021 [15]	PFQ—German version	Scores ≥ 1	Present	40%	NR	NR	71%	No partner (47%), impotent partner (15%), dyspareunia (12%), vaginal dryness (6%), and low sexual desire (6%)
İzci, F.; 2020 [2]	ASEX	Scores ranged from 5 to 30. High scores indicate high sexual dysfunction	Present Difference between groups specially in the pre-treatment phase (21.39 ± 5)	NR	NR	Desire, psychological arousal, physiological arousal, orgasm	NR	NR
Mayer, S.; 2019 [9]	SAQ and items 11 to 13 of the FSFI	High scores in the SAQ indicate more pleasure, discomfort, and higher sexual frequency than usual	Present	NR	NR	Satisfaction, discomfort, and frequency of sexual activities	BC 45.9%, OC 56.5%, and CG 76.7%	No sexual interest BC: 42.4% OC: 58.3% No partner CG: 41.7%
Buckingham, L.; 2019 [16]	PISQ	Maximum score = 48. High scores indicate good sexual function	Absent No alterations between groups Mean score = 33	NA	NA	NR	Both groups > 60%	NR
Heinzler, J.; 2018 [17]	FSFI and EORTC QLQ-CX24	FSFI scores < 26.55 indicate sexual dysfunction	Present Group mean: S1: 23.8 ± 9.7 S2: 25.3 ± 7.5	NR	3.5 (*p* = 0.0004)	Desire, arousal, satisfaction, and pain	NR	NR
Soldera, S.V.; 2018 [1]	SAQ	High scores indicate more pleasure, discomfort, and higher sexual frequency than usual	Absent Not altered compared with the CG and adjuvant therapy	NA	NA	NR	NR	NR
Corrêa, C.S.L.; 2016 [26]	FSFI	Scores < 26.0 indicate sexual dysfunction	Present CaG: (mean = 21.72)	80%		Desire, arousal, lubrication, orgasm, satisfaction, and discomfort	GCa: 40.5% GC: 75.7%	No partner CaG: 32.4% GC: 66.7%
Aerts, L.; 2015 [18]	SSFS	SSFS ≥ 5	Present (*p* < 0.01)	NR	NR	EC showed higher pain during the beginning of vaginal penetration	NR	NR
Aerts, L.; 2014 [20]	SSFS	SSFS ≥ 5	Present (*p* < 0.01)	NR	NR	Desire, arousal, and orgasm in the BCT group	NR	NR
Froeding, L.P.; 2014 [19]	FSFI, FSDS, and SVQ	FSFI < 26.55	Present	RVTG: 44.4% RAHG: 31.3%	NR	Desire RVTG:44.5% RAHG: 43.8%	RVTG:88.9% RAHG: 81.3% CG: 96.7%	NR
Aerts, L.; 2014 [20]	SSFS and SSPQ	SSFS ≥ 5	Present	NR	NR	Desire, arousal, and orgasm	VC 52% CG: NR	NR
Juraskova, I.; 2013 [27]	DSFI	Score > 16.5	Present (*p* > 0.05) CaG: Baseline: 5.41 (0.27) 6 months: 4.47 (0.37) GB: Baseline: 5.78 (0.30) 6 months: 5.42 (0.40) GPI: Baseline: 5.29 (0.52) 6 months: 4.27 (0.70)	NR	NR	Desire	CaG: 14.08% BeG: 15.27% PIG: 13.61%	NR
Abasher, S.M.; 2009 [24]	WSFQ	Scores range from 17 to 85. High scores indicate positive sexual function	Present (*p* < 0.001). Specially in patients during chemo or radiotherapy	NR	NR	Desire and satisfaction	NR	NR
Pérez, M.; 2010 [21]	Created by the authors	4-point Likert scale. High mean scores indicate more sexual dysfunction	Present Sexual function was altered in patients with mastectomy (*p* < 0.05), chemotherapy (*p* < 0.05), and radiotherapy and hormonal therapy (*p* < 0.05)	NR	2.7 (*p* = 0.0339)	Sexual interest	CDIS: 57% Stage I: 60.8% Stage IIA: 56.2% CG: 63.7%	NR
Jensen, P.T.; 2003 [22]	SVQ and UGMQ	Used in longitudinal studies	Present	NR	12 months Dyspareunia: 4.8*(95%CI: 1.4 to 16.6) Orgasm: 1.5* (95%CI: 1.1 to 2.2) Lubrication: 7.6*(95%CI: 3.2 to 18.1) Satisfaction: 2.1* (95%CI: 1.3 to 3.5)	Lubrication	CaG: 53% CG: 55%	NR
Andersen, B.L.; 1989 [23]	DSFI	> 16.5	Present	30%	NR	Desire	NR	NR

PFQ: Pelvic Floor Questionnaire; ASEX: Arizona Sexual Life Scale; SAQ: Sexual Activity Questionnaire; FSFI: Female Sexual Function Index; DSFI: Derogatis Sexual Functioning Inventory; SSFS: Short Sexual Functioning Scale; SSPQ: Specific Sexual Problems Questionnaire; EORTC QLQ-CX24: European Organization for Research and Treatment of Cancer—Quality of Life Questionnaire Cervical Cancer Module; PISQ: Pelvic Organ Prolapse/Urinary Incontinence Sexual Questionnaire; SVQ: Sexual Function-Vaginal Changes Questionnaire; UGMQ: Uro-Gynecological Morbidity Questionnaire; WSFQ: Watts Sexual Function Questionnaire; S1:with conization; S2: with dysplasia but without conization; NR: not reported; NA: not assessed; CaG: cancer group; BCT: Breast cancer treatment; BeG: benign group; PIG: pre-invasive group; RVTG: radical vaginal trachelectomy group; RAHG: hysterectomy abdominal radical group; GC: control group; VC: Vulvar cancer; EC: endometrial cancer; BC: breast cancer; OC: ovary cancer; DCIS: ductal carcinoma in situ; * Statistically significant.

**Table 4:** Methodological quality assessment according to the Quality Assessment Tool for Observational Cohort and Cross-Sectional Studies.

Study	Q1	Q2	Q3	Q4	Q5	Q6	Q7	Q8	Q9	Q10	Q11	Q12	Q13	Q14	Total
Andersen, B.L.; 1989 [23]	✓	-	✓	✓	-	✓	✓	✓	✓	NA	✓	NR	✓	✓	10
Pérez, M.; 2010 [21]	✓	✓	✓	✓	-	✓	✓	✓	✓	NA	-	NR	✓	✓	10
Froeding, L.P.; 2014 [19]	✓	✓	NR	✓	-	✓	✓	✓	✓	NA	✓	NR	-	✓	9
Jensen, P.T.; 2003 [22]	✓	✓	✓	✓	-	✓	✓	✓	✓	NA	✓	NR	-	-	9
Soldera, S.V.; 2018 [1]	✓	✓	✓	✓	-	✓	✓	NA	✓	NA	✓	NR	NA	✓	9
Baessler, K.; 2021 [15]	✓	✓	✓	-	✓	✓	✓	✓	-	NA	✓	NR	NA	-	8
Mayer, S.; 2019 [9]	✓	✓	✓	-	-	✓	✓	-	✓	NA	✓	NR	NA	✓	8
Buckingham, L.; 2019 [16]	✓	✓	-	✓	-	✓	✓	NA	✓	NA	✓	NR	-	✓	8
Heinzler, J.; 2018 [17]	✓	✓	✓	✓	-	✓	-	-	✓	NA	✓	NR	-	✓	8
Aerts, L.; 2015 [18]	✓	-	✓	✓	-	✓	✓	NA	✓	NA	✓	NR	-	✓	8
Aerts, L.; 2014 [20]	✓	✓	✓	✓	-	✓	✓	-	✓	NA	✓	NR	-	-	8
Corrêa, C.S.L.; 2016 [26]	✓	✓	NR	✓	-	✓	-	NA	✓	NA	✓	NR	NA	✓	7
Juraskova, I.; 2013 [27]	✓	-	-	NR	-	✓	-	✓	✓	NA	✓	NR	✓	✓	7
İzci, F.; 2020 [2]	✓	✓	NR	✓	-	✓	-	NA	✓	NA	✓	NR	NR	-	6
Abasher, S.M.; 2009 [24]	✓	✓	✓	✓	-	-	-	NA	-	NA	✓	NR	NA	-	5
Aerts, L.; 2014 [20]	✓	-	-	✓	-	-	-	NA	-	NA	✓	NR	NA	-	3

Q1: objective stated; Q2: population defined; Q3: participation rate ≥ 50%; Q4: sample eligibility; Q5: sample size justification; Q6: exposure prior to outcome; Q7: sufficient timeframe; Q8: levels of exposure; Q9: exposure defined and valid; Q10: exposure measured + 1; Q11: outcome defined and valid; Q12: assessors blinded; Q13: lost to follow ≤ 20%; Q14: variables adjusted; NR: not reported; NA: not applicable; ✓ denotes “Yes”; - denotes “No”.

## Data Availability

The data presented in this study are available on request from the corresponding author.
